# Chronic kidney disease onset, progression, and cardiovascular outcomes: proteomics informs biology and risk stratification

**DOI:** 10.1186/s12933-025-03049-0

**Published:** 2026-01-20

**Authors:** Jijuan Zhang, Hancheng Yu, Xingyue Song, Xianli Li, Jinchi Xie, Yuxiang Wang, Yue Li, Kun Xu, Gang Liu, Yunfei Liao, Xiong-Zhong Ruan, An Pan, Tingting Geng

**Affiliations:** 1 Department of Epidemiology and Biostatistics, Ministry of Education Key Laboratory of Environment and Health, School of Public Health, Tongji Medical College, Huazhong University of Science and Technology, Wuhan, Hubei Province China; 2 Department of Nutrition and Food Hygiene, Hubei Key Laboratory of Food Nutrition and Safety, School of Public Health, Tongji Medical College, Huazhong University of Science and Technology, Wuhan, Hubei Province China; 3 Department of Emergency, Hainan Clinical Research Center for Acute and Critical Diseases, The Second Affiliated Hospital of Hainan Medical University, Haikou, Hainan Province China; 4 Centre for Obesity and Diabetes Research, School of Public Health, Tongji Medical College, Huazhong University of Science and Technology, Wuhan, Hubei Province China; 5 Department of Endocrinology, Wuhan Union Hospital, Tongji Medical College, Huazhong University of Science and Technology, Wuhan, , Hubei Province China; 6 Centre for Lipid Research, Key Laboratory of Molecular Biology on Infectious Diseases, Ministry of Education, Chongqing Medical University, Chongqing, China

**Keywords:** Chronic kidney disease, Cardiovascular disease, Proteomics, Kidney function, End stage kidney disease

## Abstract

**Background:**

Large-scale proteomics provides an opportunity to understand chronic kidney disease (CKD) and cardiovascular disease, yet research in this field is limited. This study utilized proteomics to inform biology and risk stratification for these diseases.

**Methods:**

This cohort study included 44,779 participants free of prevalent CKD, and 3,749–4,272 participants with prevalent CKD from the UK Biobank. The Olink Explore 3072 platform quantified 2,923 plasma proteins. Cox proportional hazards models were used to assess associations of proteins with kidney diseases including CKD and end stage kidney disease, and cardiovascular diseases including coronary heart disease (CHD), stroke, and heart failure (HF). Mendelian randomization examined genetic associations, pathway analyses identified biological pathways, and predictive models were developed for incident diseases.

**Results:**

Median follow-up periods were 12.2–12.6 years. We identified 598 (20.5%) proteins shared across ≥ 2 diseases, with 595 (20.4%) showing consistent directions of associations, and 471 (16.1%) unique to a single disease. CKD and HF specifically shared the largest number of 279 (9.6%) proteins. POLR2F, TNFRSF10B, and IGFBP2 were positively associated with all five diseases, with Mendelian randomization supporting genetic associations of POLR2F with CHD and IGFBP2 with hypertensive renal disease. Pathway analyses highlighted cell adhesion, signal transduction, and cytokine-cytokine receptor interaction for disease-associated proteins. Incorporating predictive proteins into clinical models improved risk prediction for CKD, CHD, stroke, and HF, yielding Harrell’s C indices of 0.750–0.818 (corresponding increases of 0.027–0.090).

**Conclusions:**

This study deepens insights into disease biology and provides a foundation for early detection and integrated risk stratification in CKD and cardiovascular disease.

**Supplementary Information:**

The online version contains supplementary material available at 10.1186/s12933-025-03049-0.

## Research insights

### What is currently known about this topic?

- Staged progression of chronic kidney disease and cardiovascular disease share common and distinct proteomic biomarkers and pathways.
- Current clinical models exhibit suboptimal predictive performance for chronic kidney disease, end stage kidney disease, and cardiovascular disease.

### What is the key research question?

Which plasma proteins are shared or specific to chronic kidney disease, end stage kidney disease, and cardiovascular disease (including coronary heart disease, stroke, and heart failure), and to what extent do these proteins improve prediction for these diseases?

### What is new?

- 598 proteins were shared across ≥ 2 diseases and 471 were disease-specific. POLR2F, TNFRSF10B, and IGFBP2 were positively associated with all five diseases (chronic kidney disease, end stage kidney disease, coronary heart disease, stroke, and heart failure).
- Disease-associated proteins were enriched in cell adhesion, signal transduction, and cytokine-cytokine receptor interaction pathways.
- Incorporating predictive proteins into clinical models improved prediction for chronic kidney disease, coronary heart disease, stroke, and heart failure.

### How might this study influence clinical practice?

Identified proteins deepen biological insights and improve risk stratification for chronic kidney disease, end stage kidney disease, and cardiovascular disease, informing personalized prevention.

## Background

Chronic kidney disease (CKD) is a rising health issue, affecting 15–20% of adults worldwide [1]. The staged progression of CKD represents a complex and often asymptomatic continuum, beginning with normal kidney function, advancing to CKD, and eventually progressing to end stage kidney disease (ESKD) and/or various complications. In patients with CKD, cardiovascular disease (CVD) is a major health concern, affecting nearly half of patients in stages 4 and 5 [2] and remaining the leading cause of death in this population [1]. Therefore, preventing CKD and CVD is of paramount importance to improve population health and alleviate healthcare burden [3].

Staged progression of CKD and subsequent CVD are driven by multifactorial processes, such as neurohormonal activation, inflammation, and endothelial dysfunction [4, 5], that remain incompletely understood. Recent advances in proteomics have facilitated the quantification of thousands of proteins, enabling comprehensive molecular insights into CKD and CVD [6, 7]. Previous studies including the Chronic Renal Insufficiency Cohort and the Atherosclerosis Risk in Communities Cohort, have identified plasma proteins associated with kidney function decline and CKD [8–11], as well as CVD in patients with CKD [12, 13]. However, these studies are limited by small sample sizes and narrow protein profiling. Moreover, no study has systematically examined shared and disease-specific proteins in CKD and CVD. Such integrative investigation is essential for uncovering novel biomarkers, clarifying biological pathways, and enhancing predictive strategies for CKD and CVD. Besides, factors such as age, sex, and comorbidities (e.g., history of diabetes) potentially contribute to heterogeneity in CKD and CVD risk [1, 14, 15]. Clarifying their interactions with plasma proteins will deepen insights into protein-disease heterogeneity and guide precise prevention strategies.

**Fig. 1:**
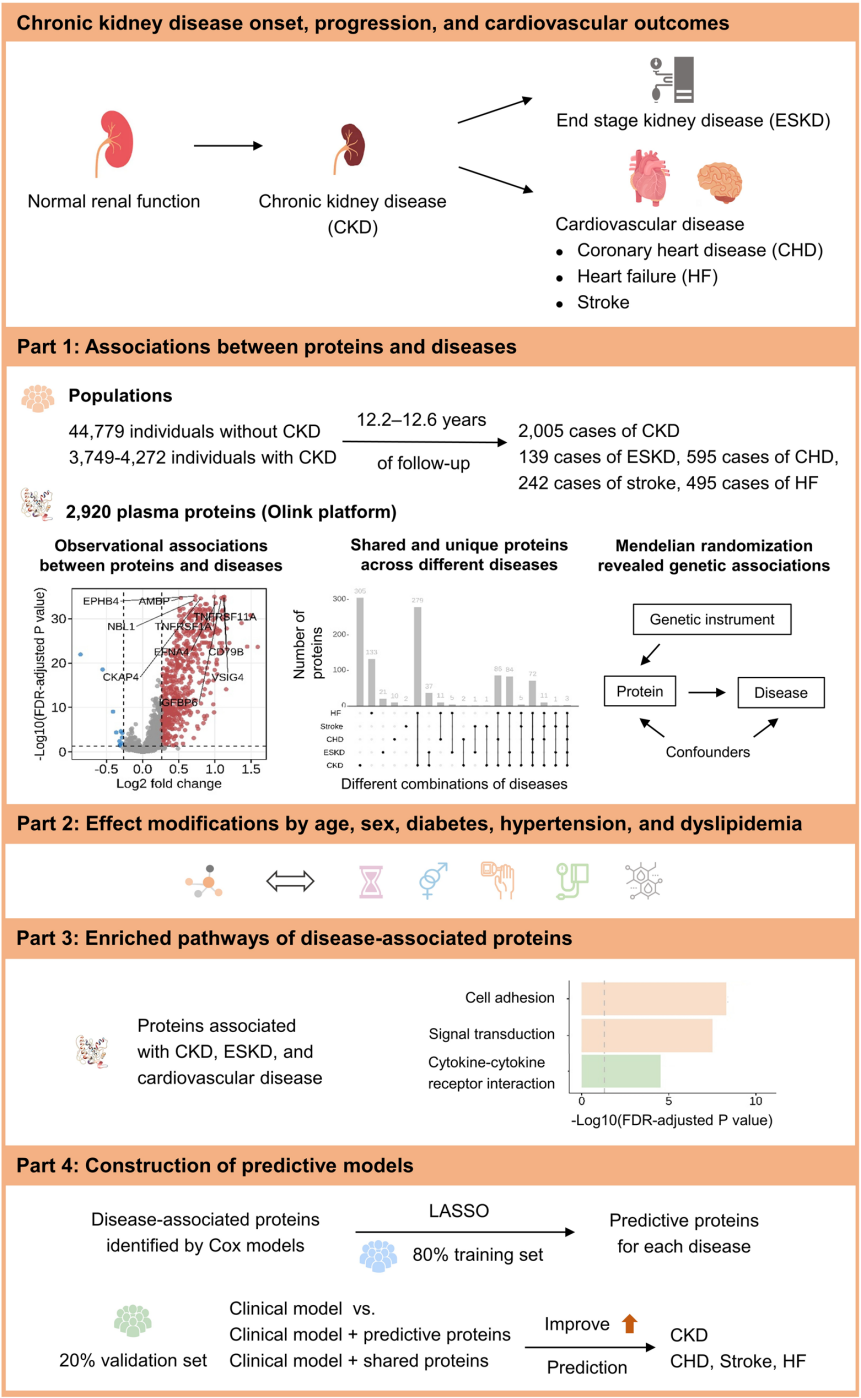
Study overview. LASSO, least absolute shrinkage and selection operator. FDR, false discovery rate

In this study, we conducted a large-scale proteomic analysis in UK Biobank participants with four aims: (1) to identify plasma proteins associated with kidney diseases including CKD and ESKD, and CVD including coronary heart disease (CHD), stroke, and heart failure (HF), highlighting shared and disease-specific proteins and evaluating potential causal relationships using Mendelian randomization (MR); (2) to assess effect modifications by age, sex, and comorbidities (hypertension, dyslipidemia, and diabetes); (3) to infer potential biological pathways of disease-associated proteins; and (4) to develop predictive models based on proteins and conventional risk factors (Fig. 1).

## Methods

### Study population

The UK Biobank is a large-scale, prospective cohort study that included more than 500,000 individuals aged 40–69 years between 2006 and 2010 [16]. The UK Biobank was approved by the North West Multi-centre Research Ethics Committee (REC reference numbers 11/NW/0382, 16/NW/0274, and 21/NW/0157), and all participants provided written informed consent. The study complies with the Declaration of Helsinki.

In this study, 44,779 participants free of CKD at baseline were included to prospectively examine associations between plasma proteins and incident CKD. For prospective associations of plasma proteins with incident ESKD, CHD, stroke, and HF, we included 3,749–4,272 participants with prevalent CKD, after excluding those with the corresponding disease at baseline. Inclusion and exclusion criteria are detailed in the Supplementary Methods, with the study flow chart in Supplementary Fig. S1.

### Plasma proteomics

The UK Biobank Pharma Proteomics Project is a collaborative consortium that profiled plasma proteomes of UK Biobank participants [17]. Details regarding proteomic assays, data processing, and quality control are available elsewhere [17]. In brief, the Olink Explore 3072 platform quantified 2,941 protein analytes representing 2,923 proteins, expressed as normalized protein expression (NPX) values. For this study, baseline proteomic data were used, excluding three proteins with > 30% missing values (NPM1, PCOLCE, GLIPR1), yielding an analytical set of 2,920 proteins (Supplementary Table S1).

### Ascertainment of outcomes

This study focused on two kidney diseases (CKD, ESKD) and three CVDs (CHD, stroke, HF). The first occurrences of diseases were identified by mapping primary care, hospital admissions, death registries, and self-reported data. Person-years were calculated from recruitment until the earliest of the first diagnosis of disease, death, loss to follow-up, or censoring (October 4, 2021). Diseases were defined using the International Classification of Diseases Tenth Edition codes (CKD: I12, I13, N03, N04, N05, N06, N08, N11, N12, N18, N19, and N25; ESKD: E85.3, N16.5, N18.0, N18.5, Q60.1, T82.4, T86.1, Y60.2, Y61.2, Y62.2, Y84.1, Z49.0-Z49.2, Z94.0, and Z99.2; CHD: I20-I25; Stroke: I60-I64; HF: I50).

### Statistical analysis

The NPX values were imputed using the mean (missing proportion 1.6–26.7%, Supplementary Table S2) and z-score standardized [17]. For covariates with ≤ 5% missingness, values were imputed using the median (continuous) or the mode (categorical). When missingness exceeded 5%, a missing indicator was used [18].

The log_2_ fold changes in protein levels were calculated for participants with and without incident disease, and group differences were assessed using t tests. Cox proportional hazards models were used to estimate hazard ratios (HRs) and 95% confidence intervals (CIs) for associations between proteins and incident diseases. The proportional hazards assumption was assessed using Schoenfeld residuals and no evident violation was found. Models were adjusted for age, sex, self-reported race, education attainment, Townsend deprivation index, body mass index, diet, physical activity, smoking status, alcohol consumption, estimated glomerular filtration rate, natural logarithm transformed urinary albumin-to-creatinine ratio, histories of hypertension, diabetes, and dyslipidemia (Supplementary Methods). When investigating associations of proteins with CKD and ESKD, we additionally adjusted for history of CVD. When examining associations between proteins and each type of CVD (CHD, stroke, and HF), we additionally adjusted for the number of the other two prevalent CVDs.

Proteins associated with at least one disease in observational analyses were evaluated for potential causal roles in cardiovascular and kidney disease. We searched the proteome-phenome atlas (https://proteome-phenome-atlas.com/), where Deng Y. et al. conducted MR analyses using protein quantitative trait loci (pQTL) as instruments to assess genetic associations between UK Biobank proteins and diverse diseases [19]. Instruments with an F-statistic < 10 were removed to reduce weak instrument bias, and those associated with more than five proteins were excluded to minimize pleiotropic effects. We reported genetic associations that were nominally significant (*P* < 0.05) and directionally consistent with observational findings.

To examine age-, sex-, diabetes-, hypertension-, and dyslipidemia-protein interactions, we performed stratified analyses by age (≤ 65 years, > 65 years), sex (men, women), histories of diabetes (yes, no), hypertension (yes, no), and dyslipidemia (yes, no). Product terms were incorporated into the models, and multiplicative interactions were evaluated with Wald tests.

To explore the biological relevance of proteins, we performed pathway enrichment analyses using Gene Ontology—biological process and Kyoto Encyclopedia of Genes and Genomes terms.

We developed predictive models for incident diseases. The dataset for each disease was randomly split into an 80% training set and a 20% internal validation set. In the training set, we constructed a least absolute shrinkage and selection operator (LASSO) penalty Cox model to select predictive proteins from those associated with each disease in observational analyses. The penalty parameter “lambda” was selected using tenfold cross-validation with the “1 standard error rule”. Clinical models included: (1) the kidney failure model, which utilized predictors from the kidney failure risk equation; (2) the extended kidney failure model, which was derived from a CKD risk equation encompassing over 5 million individuals from 34 multinational cohorts; and (3) the cardiovascular model, which utilized predictors from the Framingham CVD risk model [20–22]. Coefficients in the clinical models were refit to optimize model performance. LASSO-selected predictive proteins and proteins shared across five diseases were incorporated into the clinical models, respectively. Supplementary Table S3 summarizes model specifications. In the internal validation set, model performance was assessed using Harrell’s C index, net reclassification improvement (NRI), integrated discrimination improvement (IDI), likelihood ratio tests, calibration curves, and decision curves.

Several sensitivity analyses were conducted. Firstly, we excluded incident cases within the first year to reduce potential reverse causation bias. Secondly, we assessed protein-disease associations without imputation of missing proteomic data to account for the impact of missingness. Thirdly, we defined statistical significance for protein-disease associations using a stricter Benjamini–Hochberg false discovery rate (FDR) threshold of *P* < 0.01, and performed a permutation analysis for disease-associated proteins. Fourthly, we explored shared and disease-specific proteins across non-stroke outcomes, given the limited number of stroke-associated proteins.

Statistical analyses were performed utilizing the R software (version 4.1.3). To account for multiple comparisons, the Benjamini–Hochberg FDR correction was applied to adjust for *P*s.

## Results

### Population characteristics

The baseline characteristics of participants are presented in Supplementary Table S4. Over a median (interquartile range) follow-up of 12.6 (11.8, 13.3) years, 2,005 (4.5%) participants free of CKD at baseline developed incident CKD. In participants with baseline CKD, after median (interquartile range) follow-ups of 12.2 (11.4, 13.1) years, 12.2 (11.3, 13.1) years, 12.2 (11.4, 13.1) years, and 12.2 (11.3, 13.1) years, 139 (3.3%) participants progressed to ESKD, 595 (15.9%) developed CHD, 242 (5.7%) experienced stroke, and 495 (11.6%) developed HF, respectively.

### Associations of proteins with kidney and cardiovascular diseases

**Fig. 2:**
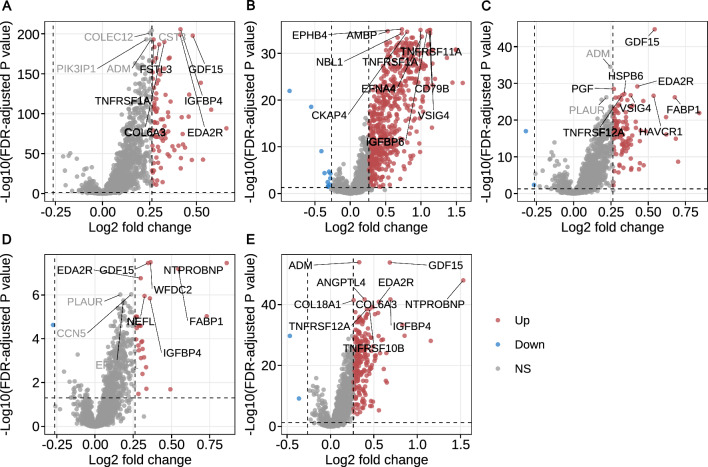
Volcano plot showing the log_2_ fold changes in plasma protein levels between participants with and without incident disease. **A** chronic kidney disease. **B** end stage kidney disease. **C** coronary heart disease. **D** stroke. **E** heart failure. The text in the figure represents the top 10 proteins of each disease, sorted by FDR-corrected *P *from t tests. The proteins lying above the horizontal dotted line have the FDR-corrected *P* < 0.05. Red/blue dots mark proteins with log_2_ fold change > log_2_(1.2) or <  − log_2_(1.2). FDR, false discovery rate. NS, not significant

**Fig. 3:**
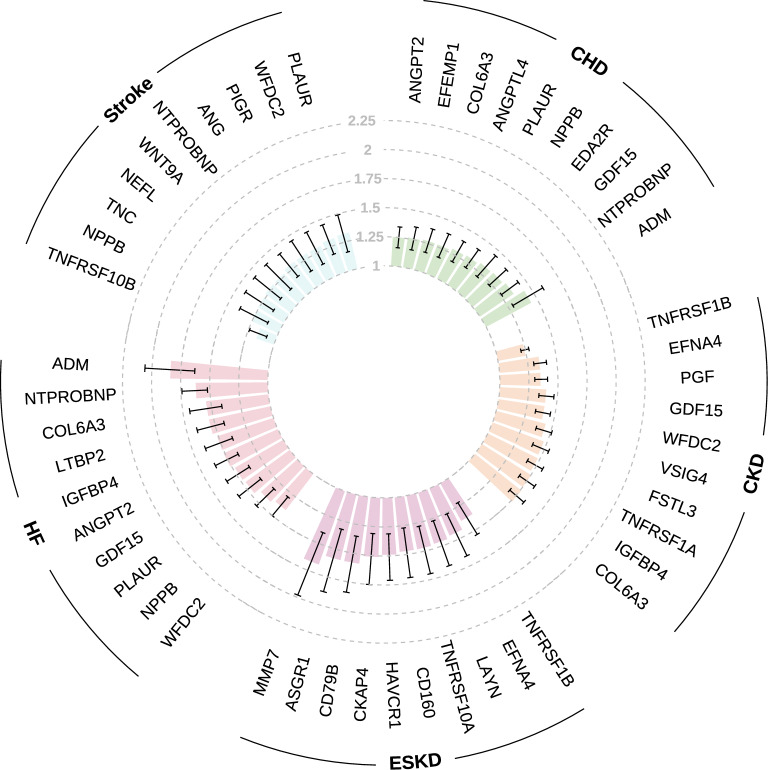
The top 10 proteins selected by FDR-corrected *P* for each disease. Bars represent hazard ratios, with error bars indicating 95% confidence intervals. FDR, false discovery rate. CHD, coronary heart disease. CKD, chronic kidney disease. ESKD, end stage kidney disease. HF, heart failure

The log_2_ fold changes in protein levels between participants with and without incident disease are shown in Fig. 2. In multivariable-adjusted Cox proportional hazards models, 886 (30.3%) proteins were associated with incident CKD, 224 (7.7%) with ESKD, 195 (6.7%) with CHD, 24 (0.8%) with stroke, and 690 (23.6%) with HF (Supplementary Table S5). The top 10 proteins for each disease, ranked by FDR-corrected *P* from Cox proportional hazards models, are presented in Fig. 3 and were all positively associated with the corresponding diseases.

Sensitivity analyses demonstrated robust results when excluding incident cases within the first year (Supplementary Table S6), assessing protein-disease associations without imputation of missing proteomic data (Supplementary Table S7), using an FDR-corrected *P* < 0.01 to define statistical significance (Supplementary Table S8), or performing permutation analysis (Supplementary Table S9).

### Shared and disease-specific proteomic profiles

**Fig. 4:**
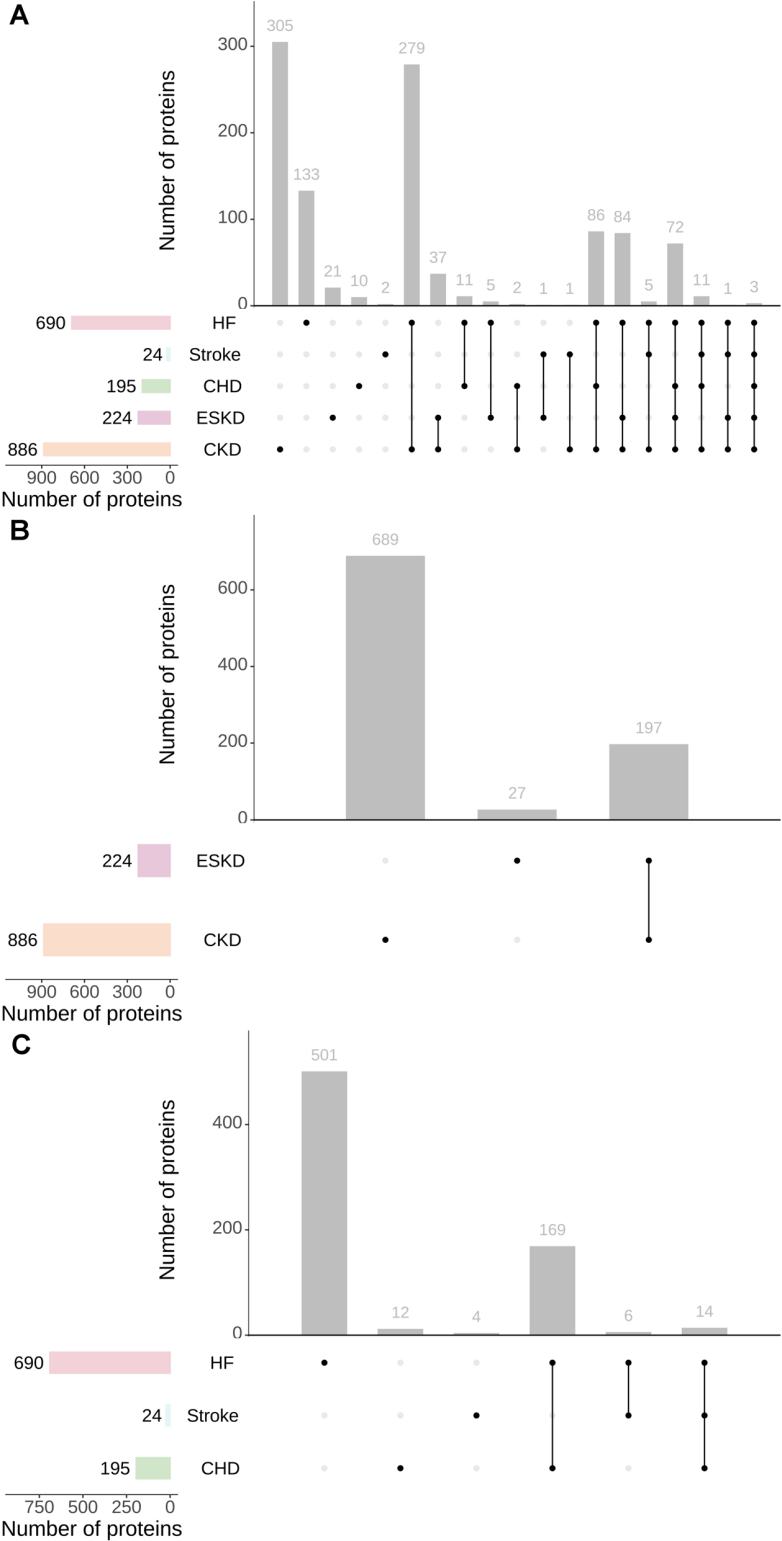
Upset plots of shared and disease-specific proteins across **A** five diseases, **B** chronic kidney disease and end stage kidney disease, and **C** cardiovascular diseases. CHD, coronary heart disease. CKD, chronic kidney disease. ESKD, end stage kidney disease. HF, heart failure

Among 2,920 proteins, 1,069 (36.6%) were associated with at least one disease. A total of 598 (20.5%) proteins were associated with at least two diseases, of which 570 (19.5%) showed consistent positive and 25 (0.9%) consistent negative associations (Supplementary Table S5). Three (0.1%) proteins, POLR2F, TNFRSF10B, and IGFBP2 were positively associated with all five diseases (Fig. 4A). CKD and HF specifically shared the largest number of 279 (9.6%) proteins (Fig. 4A). In sensitivity analyses, 75 (2.6%) proteins were shared across all non-stroke outcomes (Supplementary Table S10 and Supplementary Fig. S2).

As for kidney outcomes, 197 (6.7%) proteins were positively associated with both CKD and ESKD (Fig. 4B, Supplementary Table S11). For cardiovascular outcomes, 14 (0.5%) proteins were positively associated with CHD, stroke, and HF (Fig. 4C, Supplementary Table S12).

Meanwhile, 471 (16.1%) proteins were specifically associated with a single disease, including 305 (10.4%) for CKD, 21 (0.7%) for ESKD, 10 (0.3%) for CHD, 2 (0.1%) for stroke, and 133 (4.6%) for HF (Fig. 4A). Ranked by FDR-corrected *P*, the top disease-specific proteins were EFCAB14 for CKD, RAB44 for ESKD, ATXN2 for CHD, PNLIP for stroke, and SFTPD for HF (Supplementary Tables S13).

### Genetic associations revealed by Mendelian randomization

Among the 1,069 (36.6%) proteins associated with at least one disease in observational analyses, 309 (10.6%) exhibited genetic associations with kidney diseases, CHD, or HF, with directions consistent with the observational findings (Supplementary Table S14). Specifically, 252 (8.6%) proteins were genetically associated with kidney diseases, 39 (1.3%) with CHD, and 38 (1.3%) with HF. Notably, POLR2F and IGFBP2 were observationally associated with five diseases, and MR further supported the genetic association of POLR2F with CHD and IGFBP2 with hypertensive renal disease.

### Effect modifications by age, sex, and morbidities

**Fig. 5:**
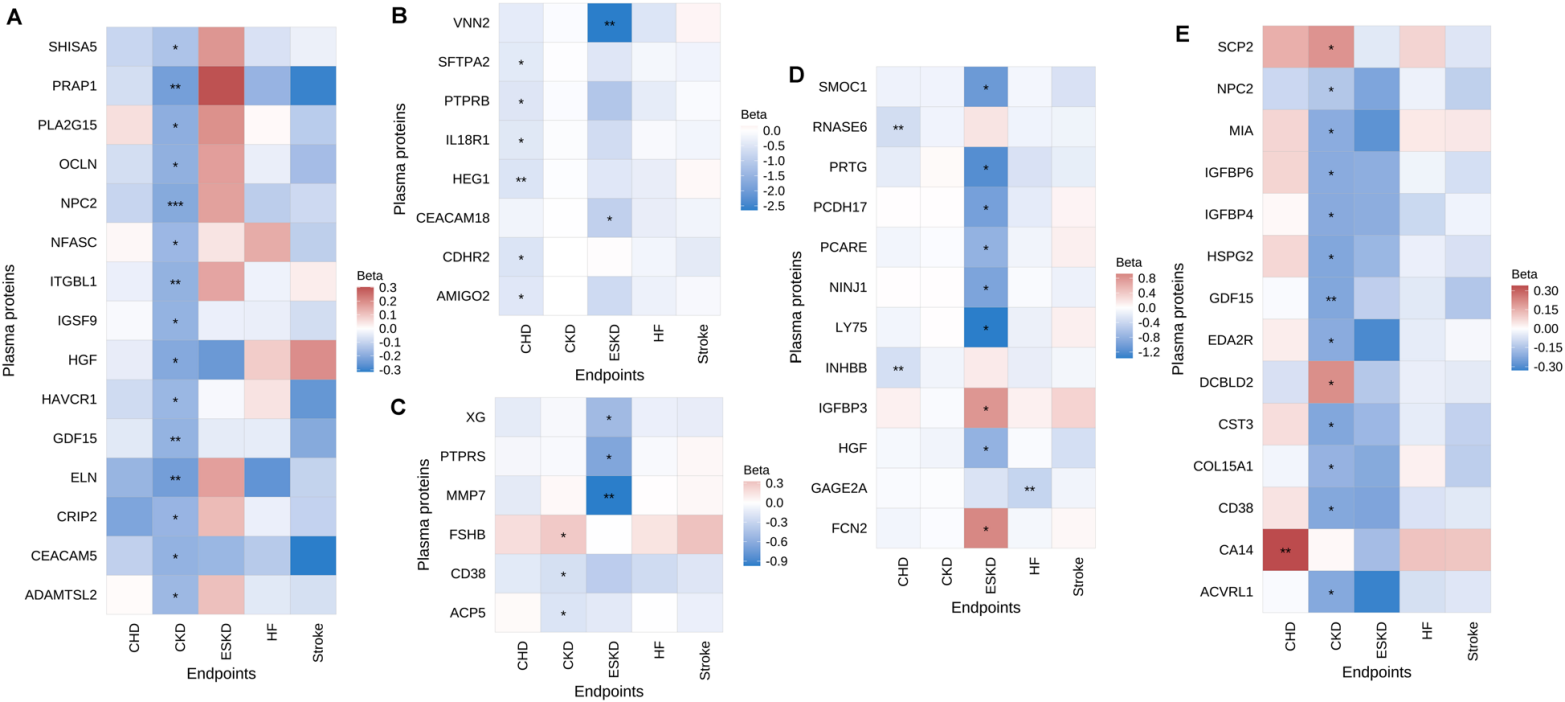
Age-, sex-, diabetes-, hypertension-, and dyslipidemia-protein interactions for five diseases. **A** age-protein interactions. **B** hypertension-protein interactions. **C** sex-protein interactions. **D** dyslipidemia-protein interactions. **E** diabetes-protein interactions. Heatmap was plotted based on *β* estimates derived from an age/sex/diabetes/hypertension/dyslipidemia-protein product term in the models. Proteins with significant interactions with at least one disease were included in the heatmap. Heatmap color of red indicates a stronger association within men, participants > 65 years, or participants with diabetes/hypertension/dyslipidemia, whereas blue indicates opposite. ***FDR-adjusted *P*-interaction < 0.001; **FDR-adjusted *P*-interaction < 0.01; *FDR-adjusted *P*-interaction < 0.05. CHD, coronary heart disease. CKD, chronic kidney disease. ESKD, end stage kidney disease. HF, heart failure. FDR, false discovery rate

A total of 15 (0.5%) proteins exhibited interactions with age on CKD risk (Fig. 5A), while 6 (0.2%) interacted with sex, 14 (0.5%) with diabetes, 8 (0.3%) with hypertension, and 12 (0.4%) with dyslipidemia (all FDR-corrected *P* < 0.05). Specifically, hypertension history interacted with 6 proteins on CHD risk and 2 on ESKD risk (Fig. 5B). Sex modified the associations of 3 proteins with CKD risk and 3 with ESKD risk (Fig. 5C). Dyslipidemia history interacted with 2, 1, and 9 proteins on CHD, HF, and ESKD risks, respectively (Fig. 5D). Diabetes history interacted with 1 protein on CHD risk and 13 on CKD risk (Fig. 5E).

### Enriched pathways

Proteins associated with CKD, ESKD, or CVD were primarily involved in cell adhesion, signal transduction, and cytokine-cytokine receptor interaction pathways (all FDR-corrected *P* < 0.05; Supplementary Figs. S3–7).

### Predictive performance of models

In training sets, the LASSO models selected 34 (1.2%) predictive proteins for CKD, 9 (0.3%) for ESKD, 29 (1.0%) for CHD, 11 (0.4%) for stroke, and 19 (0.7%) for HF (Supplementary Table S15). In internal validation sets, adding LASSO-selected predictive proteins to clinical models improved prediction of CKD, CHD, HF, and stroke (all *P* for likelihood ratio tests < 0.05), yielding Harrell’s C indices of 0.750–0.818 (corresponding increases of 0.027–0.090), continuous NRIs of 0.318–0.784, categorical NRIs of 0.079–0.316, and IDIs of 0.032–0.114 (Table 1). Incorporating the three proteins shared across all five diseases in observational analyses (POLR2F, TNFRSF10B, and IGFBP2) into the traditional risk factor model enhanced prediction of CKD and HF, yielding Harrell’s C indices of 0.744–0.812 (corresponding increases of 0.009–0.045), continuous NRIs of 0.279–0.475, categorical NRIs of 0.039–0.182, and IDIs of 0.005 to 0.036 (Table 1). The models retained predictive ability at 5, 10, and 15 years, though the extent and pattern of predictive improvement differed across diseases (Supplementary Table S16). The predictive improvement for CKD, stroke, and HF was greater at earlier time points, whereas that for CHD was more pronounced at later time points. Calibration curves indicated adequate predictive accuracy of the models for CKD, CHD, stroke, and HF (Supplementary Figs. S8-9). The clinical models, as well as their combinations with predictive proteins or with the three shared proteins, demonstrated net benefits (Supplementary Figs. S10–11).

**Table 1:** Predictive performance of various models for incident diseases in the internal validation sets.^a^

	Harrell’s C-index	*P*^b^	Continuous NRI	Categorical NRI	IDI	*P*^c^
*Chronic kidney disease*						
KF model	0.783 (0.760, 0.806)	Reference	Reference	Reference	Reference	Reference
Extended KF model	0.808 (0.786, 0.830)	< 0.001	0.332 (0.220, 0.445)	0.094 (0.047, 0.136)	0.021 (0.013, 0.030)	< 0.001
Extended KF model + predicted proteins	0.818 (0.796, 0.839)	< 0.001	0.362 (0.248, 0.474)	0.092 (0.041, 0.145)	0.042 (0.029, 0.055)	< 0.001
KF model + predicted proteins	0.810 (0.788, 0.832)	< 0.001	0.318 (0.210, 0.431)	0.079 (0.027, 0.128)	0.036 (0.025, 0.047)	< 0.001
Extended KF model + shared proteins	0.812 (0.791, 0.833)	< 0.001	0.435 (0.321, 0.548)	0.099 (0.052, 0.149)	0.027 (0.017, 0.037)	< 0.001
KF model + shared proteins	0.792 (0.770, 0.814)	< 0.001	0.279 (0.164, 0.383)	0.039 (0.014, 0.066)	0.005 (0.003, 0.008)	< 0.001
*End stage kidney disease*						
KF model	0.942 (0.908, 0.977)	Reference	Reference	Reference	Reference	Reference
Extended KF model	0.943 (0.907, 0.979)	0.449	0.226 (-0.179, 0.637)	0.051 (-0.087, 0.188)	-0.011 (-0.065, 0.047)	0.214
Extended KF model + predicted proteins	0.936 (0.893, 0.978)	0.687	0.316 (-0.071, 0.731)	0.064 (-0.087, 0.213)	-0.035 (-0.121, 0.055)	< 0.001
KF model + predicted proteins	0.938 (0.897, 0.979)	0.631	0.143 (-0.282, 0.535)	0.047 (-0.112, 0.207)	-0.011 (-0.089, 0.073)	< 0.001
Extended KF model + shared proteins	0.950 (0.918, 0.981)	0.188	0.496 (0.056, 0.931)	0.125 (0.056, 0.240)	0.010 (-0.058, 0.094)	0.114
KF model + shared proteins	0.948 (0.917, 0.980)	0.105	0.439 (0.004, 0.859)	0.056 (0.033, 0.080)	0.031 (-0.011, 0.069)	0.071
*Coronary heart disease*						
CV model	0.707 (0.665, 0.749)	Reference	Reference	Reference	Reference	Reference
CV model + predicted proteins	0.756 (0.718, 0.795)	< 0.001	0.505 (0.283, 0.707)	0.175 (0.053, 0.297)	0.070 (0.039, 0.102)	< 0.001
CV model + shared proteins	0.722 (0.681, 0.763)	0.039	0.349 (0.138, 0.552)	0.034 (-0.042, 0.116)	0.012 (-0.004, 0.025)	0.004
*Stroke*						
CV model	0.676 (0.614, 0.738)	Reference	Reference	Reference	Reference	Reference
CV model + predicted proteins	0.750 (0 .684, 0.816)	0.005	0.360 (0.047, 0.651)	0.167 (-0.009, 0.354)	0.032 (0.011, 0.056)	0.002
CV model + shared proteins	0.701 (0.638, 0.764)	0.106	0.269 (-0.043, 0.584)	0.016 (-0.147, 0.192)	0.012 (0.000, 0.024)	0.008
*Heart failure*						
CV model	0.699 (0.648, 0.750)	Reference	Reference	Reference	Reference	Reference
CV model + predicted proteins	0.789 (0.744, 0.834)	< 0.001	0.784 (0.555, 1.012)	0.316 (0.179, 0.447)	0.114 (0.073, 0.158)	< 0.001
CV model + shared proteins	0.744 (0.695, 0.794)	< 0.001	0.475 (0.231, 0.703)	0.182 (0.065, 0.294)	0.036 (0.009, 0.059)	< 0.001

^a^The NRI and IDI were calculated over a 10-year period. CV, cardiovascular; KF, kidney failure; NRI, net reclassification improvement; IDI, integrated discrimination improvement. The predicted proteins were selected by least absolute shrinkage and selection operator models. The shared proteins are POLR2F, TNFRSF10B, and IGFBP2, which are significantly associated with all five diseases (chronic kidney disease, end stage kidney disease, coronary heart disease, stroke, and heart failure)

^b^*P* values from Student’s t tests assessing whether the model’s C-index exceeds that of the reference model

^c^*P* values for model comparison were obtained using likelihood ratio tests

## Discussion

This study comprehensively examined associations of 2,920 plasma proteins with the risks of CKD, ESKD, CHD, stroke, and HF. We identified 598 proteins shared across at least two diseases and 471 proteins unique to a single disease. POLR2F, TNFRSF10B, and IGFBP2 were positively associated with all five diseases, with MR further supporting genetic associations of POLR2F with CHD, and IGFBP2 with hypertensive renal disease. Proteins associated with CKD, ESKD, or CVD were primarily enriched in cell adhesion, signal transduction, and cytokine-cytokine receptor interaction pathways. Additionally, incorporating predictive or shared proteins into clinical models significantly enhanced disease risk prediction. Collectively, these findings underscore the value of proteomic profiling for informing disease biology and advancing early detection and integrated risk stratification of CKD and CVD.

Among proteins associated with CKD or ESKD, well-known biomarkers of kidney function were identified, such as CST3, FABP1, HAVCR1 (KIM-1), IGFBP7, LCN2 (NGAL), and UMOD [23], suggesting the reliability of our proteomic approach. We identified 689 proteins specifically associated with CKD rather than ESKD, suggesting a potential role in the early stage of kidney disease. On the other hand, 27 proteins were specifically associated with ESKD, likely contributing to the late-stage pathophysiology. Notably, 197 proteins were shared across CKD and ESKD, indicating a continuous role throughout staged progression of kidney disease. Among them, members of the insulin-like growth factor binding protein family (IGFBP1, IGFBP2, IGFBP4, and IGFBP6) bind and regulate IGF-1/IGF-2 stability and signaling [24], thereby influencing podocyte function and apoptosis, and contributing to the pathogenesis and progression of CKD [25–28]. Several members of the tumor necrosis factor receptor superfamily are implicated in both CKD and ESKD. Among them, TNFRSF10A, TNFRSF10B, TNFRSF1A, TNFRSF21 assembles the death-inducing signaling complex, activating caspase-8 and triggering apoptosis. Receptors such as TNFRSF1B, TNFRSF11A, TNFRSF12A, TNFRSF13B, TNFRSF14, TNFRSF19, TNFRSF4, TNFRSF8, and TNFRSF9 primarily bind to tumor necrosis factor receptor-associated factors to regulate immune and inflammatory responses. TNFRSF6B functions as a decoy receptor to block pro-apoptotic and pro-inflammatory pathways [29]. Collectively, these proteins may represent potential biomarkers or targets across staged kidney disease progression [30–32].

In participants with CKD, established cardiovascular biomarkers such as NTproBNP, SDC4, IL6, TNF, and GDF15 were identified [33, 34], supporting the validity of our protein findings. For CVD, 501 proteins were specifically associated with HF, 12 with CHD, and 4 with stroke. These disease-specific proteins may capture distinct pathophysiological processes, supporting disease-specific monitoring and targeted interventions. Importantly, 14 proteins were shared across CHD, stroke, and HF. These shared proteins likely reflect systemic pathways such as inflammation, endothelial dysfunction, and metabolic dysregulation that drive cardiovascular pathology [35–37], thereby capturing overall cardiovascular vulnerability and offering potential value for global risk assessment. Among them, ANG is a potent angiogenic factor implicated in atherosclerosis and has been proposed as a risk marker for major cardiovascular events [38, 39]. Circulating PLAUR, primarily generated by enzymatic cleavage of its membrane-bound form on immune cells, promotes chronic inflammation and atherosclerosis and thus represents a promising therapeutic target for CVD [40]. WFDC2 primarily modulates extracellular matrix degradation, driving collagen deposition and fibrosis that contribute to ventricular remodeling and vascular stiffness, thereby positioning it as a potential therapeutic target for antifibrotic strategies [41, 42].

Staged progression of CKD and CVD involve overlapping pathophysiological processes [4, 5]. Identifying proteins shared across CKD and CVD therefore holds promise for uncovering broad-spectrum biomarkers or targets for multiple diseases. In this study, POLR2F, TNFRSF10B, and IGFBP2 showed positive associations with all five diseases. TNFRSF10B is a mediator of apoptotic signaling, and has been implicated in cerebral endothelial cell apoptosis, blood–brain barrier dysfunction, angiogenic impairment [43], and kidney function decline [10]. IGFBP2, primarily through regulating IGF-1/IGF-2 stability and signaling, has been linked to glucose-lipid metabolism, ventricular and vascular remodeling, and podocyte apoptosis [26, 44, 45]. These lines of evidence suggested TNFRSF10B and IGFBP2 as potential biomarkers or targets for both kidney and cardiovascular health. Notably, POLR2F functions as a DNA-dependent RNA polymerase and remains relatively understudied. Its expression in cardiac, vascular, and kidney tissues suggests a possible role in transcriptional regulation within these organs [46].

Effect modification analyses revealed interactions between proteins and key factors such as age, sex, and metabolic morbidities, emphasizing the need to consider subgroup differences in protein-disease associations, providing a basis for tailored prevention strategies.

Understanding the biological functions of proteomic biomarkers provides mechanistic insights into diseases, and evaluating their predictive value further advances their potential for clinical application. In this study, incorporating predictive or shared proteins into clinical models enhanced prediction for CKD, CHD, stroke, and HF (C indices 0.701**–**0.818), and markedly for ESKD (C indices 0.936–0.950). These findings are comparable to previous reports [11–13], though the high C-index for ESKD may reflect the low event rate, which can inflate discrimination metrics. Overall, these findings suggest the clinical utility of protein-based risk stratification for early detection of CKD and CVD, and efficient resource allocation.

Compared with previous studies examining proteomic profiles of kidney function decline or CKD [8–11], or those investigating proteomic signatures of CVD in patients with CKD [12, 13], this study innovatively adopted a holistic approach by integrating proteomic features of CKD onset, progression, and CVD. Our analysis systematically revealed shared and disease-specific proteins, offering a concise perspective on common and divergent processes underlying the cardio-kidney continuum. The large sample size, prospective design, and substantial number of measured proteins strengthened the research quality and the robustness of the findings.

However, several limitations should be noted. Firstly, while the Olink platform was used to quantify plasma proteins, it does not encompass the entire human proteome. Secondly, protein measurements in this study were based on relative quantification, which may restrict the applicability of proteomic predictive models to external populations. Thirdly, the predictive models have not been externally validated due to the lack of an independent dataset, although internal validation yielded robust results. Therefore, future studies particularly those utilizing alternative proteomic platforms (e.g., SomaScan) and absolute quantification are needed to validate our findings and facilitate clinical translation. Fourthly, this study predominantly included white participants, so caution is warranted when generalizing findings to other populations. Finally, the current MR results are limited by potential weak instruments, possible pleiotropy from trans-pQTLs, nominal significance without multiple-testing correction, and reliance on publicly available summary statistics that precluded independent sensitivity analyses. Therefore, the MR findings should be regarded as exploratory and complementary to observational associations rather than definitive causal evidence.

## Conclusions

In conclusion, this large-scale proteomic study highlighted potential biomarkers or targets for CKD and CVD, such as POLR2F, TNFRSF10B, and IGFBP2. The disease-associated proteins were primarily involved in cell adhesion, signal transduction, and cytokine-cytokine receptor interaction pathways. Additionally, proteomic predictive models enhanced risk stratification beyond traditional clinical models. Taken together, these findings deepen insights into disease biology and provide a foundation for early detection and integrated risk stratification in CKD and CVD. Future studies across diverse populations, employing alternative proteomic platforms (e.g., SomaScan) and absolute quantification, will be essential to confirm these findings.

## Supplementary Information

Additional file 1

Additional file 2

## Data Availability

The UK Biobank is an open-access database. This study has been conducted using the UK Biobank resource under application number 88159.

## Abbreviations

CHD: Coronary heart disease
CI: Confidence interval
CKD: Chronic kidney disease
CVD: Cardiovascular disease
ESKD: End stage kidney disease
FDR: False discovery rate
HR: Hazard ratio
HF: Heart failure
IDI: Integrated discrimination improvement
LASSO: Least absolute shrinkage and selection operator
NPX: Normalized protein expression
NRI: Net reclassification improvement
pQTL: Protein quantitative trait loci

## References

[1] Matsushita K, Ballew SH, Wang AY, Kalyesubula R, Schaeffner E, Agarwal R. Epidemiology and risk of cardiovascular disease in populations with chronic kidney disease. Nat Rev Nephrol. 2022;18(11):696–707. 36104509 10.1038/s41581-022-00616-6

[2] Stevens PE, O’Donoghue DJ, de Lusignan S, Van Vlymen J, Klebe B, Middleton R, et al. Chronic kidney disease management in the United Kingdom: neoerica project results. Kidney Int. 2007;72(1):92–9. 17440495 10.1038/sj.ki.5002273

[3] Webster AC, Nagler EV, Morton RL, Masson P. Chronic Kidney Disease. Lancet. 2017;389(10075):1238–52. 27887750 10.1016/S0140-6736(16)32064-5

[4] Rangaswami J, Bhalla V, Blair JEA, Chang TI, Costa S, Lentine KL, et al. Cardiorenal syndrome: classification, pathophysiology, diagnosis, and treatment strategies: a scientific statement from the American Heart Association. Circulation. 2019;139(16):e840–78. 30852913 10.1161/CIR.0000000000000664

[5] Zhao BR, Hu XR, Wang WD, Zhou Y. Cardiorenal syndrome: clinical diagnosis, molecular mechanisms and therapeutic strategies. Acta Pharmacol Sin. 2025. 10.1038/s41401-025-01476-z. 39910210 10.1038/s41401-025-01476-z PMC12098865

[6] Dubin RF, Rhee EP. Proteomics and metabolomics in kidney disease, including insights into etiology, treatment, and prevention. Clin J Am Soc Nephrol. 2020;15(3):404–11. 31636087 10.2215/CJN.07420619 PMC7057308

[7] Tracz J, Luczak M. Applying proteomics and integrative “omics” strategies to decipher the chronic kidney disease-related atherosclerosis. Int J Mol Sci. 2021. 10.3390/ijms22147492. 34299112 10.3390/ijms22147492 PMC8305100

[8] Grams ME, Surapaneni A, Chen J, Zhou L, Yu Z, Dutta D, et al. Proteins associated with risk of kidney function decline in the general population. J Am Soc Nephrol. 2021;32(9):2291–302. 34465608 10.1681/ASN.2020111607 PMC8729856

[9] Matías-García PR, Wilson R, Guo Q, Zaghlool SB, Eales JM, Xu X, et al. Plasma proteomics of renal function: a transethnic meta-analysis and Mendelian randomization study. J Am Soc Nephrol. 2021;32(7):1747–63. 34135082 10.1681/ASN.2020071070 PMC8425654

[10] Lin JS, Nano J, Petrera A, Hauck SM, Zeller T, Koenig W, et al. Proteomic profiling of longitudinal changes in kidney function among middle-aged and older men and women: the KORA S4/F4/FF4 study. BMC Med. 2023;21(1):245. 37407978 10.1186/s12916-023-02962-z PMC10324145

[11] Dubin RF, Deo R, Ren Y, Wang J, Zheng Z, Shou H, et al. Proteomics of CKD progression in the chronic renal insufficiency cohort. Nat Commun. 2023;14(1):6340. 37816758 10.1038/s41467-023-41642-7 PMC10564759

[12] Dubin RF, Deo R, Ren Y, Wang J, Pico AR, Mychaleckyj JC, et al. Incident heart failure in chronic kidney disease: proteomics informs biology and risk stratification. Eur Heart J. 2024;45(30):2752–67. 38757788 10.1093/eurheartj/ehae288 PMC11313584

[13] Deo R, Dubin RF, Ren Y, Murthy AC, Wang J, Zheng H, et al. Proteomic cardiovascular risk assessment in chronic kidney disease. Eur Heart J. 2023;44(23):2095–110. 37014015 10.1093/eurheartj/ehad115 PMC10281556

[14] Ku E, Inker LA, Tighiouart H, McCulloch CE, Adingwupu OM, Greene T, et al. Angiotensin-converting enzyme inhibitors or angiotensin-receptor blockers for advanced chronic kidney disease : a systematic review and retrospective individual participant-level meta-analysis of clinical trials. Ann Intern Med. 2024;177(7):953–63. 38950402 10.7326/M23-3236 PMC12519637

[15] Heerspink HJL, Jongs N, Chertow GM, Langkilde AM, McMurray JJV, Correa-Rotter R, et al. Effect of dapagliflozin on the rate of decline in kidney function in patients with chronic kidney disease with and without type 2 diabetes: a prespecified analysis from the DAPA-CKD trial. Lancet Diabetes Endocrinol. 2021;9(11):743–54. 34619108 10.1016/S2213-8587(21)00242-4

[16] Sudlow C, Gallacher J, Allen N, Beral V, Burton P, Danesh J, et al. UK biobank: an open access resource for identifying the causes of a wide range of complex diseases of middle and old age. PLoS Med. 2015;12(3):e1001779. 25826379 10.1371/journal.pmed.1001779 PMC4380465

[17] Sun BB, Chiou J, Traylor M, Benner C, Hsu YH, Richardson TG, et al. Plasma proteomic associations with genetics and health in the UK Biobank. Nature. 2023;622(7982):329–38. 37794186 10.1038/s41586-023-06592-6 PMC10567551

[18] Zhang JJ, Yu HC, Geng TT, Zhang JJ, Zhou XT, Wang YX, et al. Serum 25-hydroxyvitamin D concentrations, vitamin D receptor polymorphisms, and risk of infections among individuals with type 2 diabetes: a prospective cohort study. Am J Clin Nutr. 2024;120(2):398–406. 38914226 10.1016/j.ajcnut.2024.06.007

[19] Deng YT, You J, He Y, Zhang Y, Li HY, Wu XR, et al. Atlas of the plasma proteome in health and disease in 53,026 adults. Cell. 2025;188(1):253-71.e7. 39579765 10.1016/j.cell.2024.10.045

[20] Tangri N, Grams ME, Levey AS, Coresh J, Appel LJ, Astor BC, et al. Multinational assessment of accuracy of equations for predicting risk of kidney failure: a meta-analysis. JAMA. 2016;315(2):164–74. 26757465 10.1001/jama.2015.18202 PMC4752167

[21] Nelson RG, Grams ME, Ballew SH, Sang Y, Azizi F, Chadban SJ, et al. Development of risk prediction equations for incident chronic kidney disease. JAMA. 2019;322(21):2104–14. 31703124 10.1001/jama.2019.17379 PMC6865298

[22] D’Agostino RB Sr, Vasan RS, Pencina MJ, Wolf PA, Cobain M, Massaro JM, et al. General cardiovascular risk profile for use in primary care: the Framingham Heart Study. Circulation. 2008;117(6):743–53. 18212285 10.1161/CIRCULATIONAHA.107.699579

[23] Zhang WR, Parikh CR. Biomarkers of acute and chronic kidney disease. Annu Rev Physiol. 2019;81:309–33. 30742783 10.1146/annurev-physiol-020518-114605 PMC7879424

[24] Choi E, Duan C, Bai XC. Regulation and function of insulin and insulin-like growth factor receptor signalling. Nat Rev Mol Cell Biol. 2025;26(7):558–80. 39930003 10.1038/s41580-025-00826-3 PMC12631569

[25] Lay AC, Hale LJ, Stowell-Connolly H, Pope RJP, Nair V, Ju W, et al. IGFBP-1 expression is reduced in human type 2 diabetic glomeruli and modulates β1-integrin/FAK signalling in human podocytes. Diabetologia. 2021;64(7):1690–702. 33758952 10.1007/s00125-021-05427-1 PMC8187213

[26] Wang X, Zhang Y, Chi K, Ji Y, Zhang K, Li P, et al. IGFBP2 induces podocyte apoptosis promoted by mitochondrial damage via integrin α5/FAK in diabetic kidney disease. Apoptosis. 2024;29(7–8):1109–25. 38796567 10.1007/s10495-024-01974-1

[27] Aylward RE, Hayward S, Chesnaye NC, Janse RJ, Jonsson PA, Torino C, et al. Cardiometabolic protein expression levels and pathways associated with kidney function decline in older European adults with advanced kidney disease. Clin Kidney J. 2025;18(4):079. 10.1093/ckj/sfaf079 PMC12059643 40342947

[28] Liang J, Huang Y, Peng D, Xie Y, Liu Y, Lu X, et al. IGFBP2 and IGFBP4 interact to activate complement pathway in diabetic kidney disease. Ren Fail. 2025;47(1):2440528. 39806768 10.1080/0886022X.2024.2440528 PMC11734388

[29] Xu J. The role of tumor necrosis factor receptor superfamily in cancer: insights into oncogenesis, progression, and therapeutic strategies. NPJ Precis Oncol. 2025;9(1):275. 40770489 10.1038/s41698-025-00990-x PMC12328804

[30] Artinger K, Kirsch AH, Mooslechner AA, Cooper DJ, Aringer I, Schuller M, et al. Blockade of tumor necrosis factor superfamily members CD30 and OX40 abrogates disease activity in murine immune-mediated glomerulonephritis. Kidney Int. 2021;100(2):336–48. 33785369 10.1016/j.kint.2021.02.039

[31] Tseng WC, Yang WC, Yang AH, Hsieh SL, Tarng DC. Expression of TNFRSF6B in kidneys is a novel predictor for progression of chronic kidney disease. Mod Pathol. 2013;26(7):984–94. 23449012 10.1038/modpathol.2013.29

[32] He J, Liu X, Lu J, Hu H, Quan M, Chen Y, et al. Bruceine A derivative P1 alleviates renal inflammation via Tnfrsf12a pathway in diabetic nephropathy. Int Immunopharmacol. 2025;165:115419. 40902397 10.1016/j.intimp.2025.115419

[33] Xu C, Tsihlis G, Chau K, Trinh K, Rogers NM, Julovi SM. Novel perspectives in chronic kidney disease-specific cardiovascular disease. Int J Mol Sci. 2024. 10.3390/ijms25052658. 38473905 10.3390/ijms25052658 PMC10931927

[34] Schulte C, Barwari T, Joshi A, Zeller T, Mayr M. Noncoding RNAs versus protein biomarkers in cardiovascular disease. Trends Mol Med. 2020;26(6):583–96. 32470385 10.1016/j.molmed.2020.02.001

[35] Del Pinto R, Ferri C. Inflammation-accelerated senescence and the cardiovascular system: mechanisms and perspectives. Int J Mol Sci. 2018. 10.3390/ijms19123701. 30469478 10.3390/ijms19123701 PMC6321367

[36] Xu S, Ilyas I, Little PJ, Li H, Kamato D, Zheng X, et al. Endothelial dysfunction in atherosclerotic cardiovascular diseases and beyond: from mechanism to pharmacotherapies. Pharmacol Rev. 2021;73(3):924–67. 34088867 10.1124/pharmrev.120.000096

[37] Pasut A, Lama E, Van Craenenbroeck AH, Kroon J, Carmeliet P. Endothelial cell metabolism in cardiovascular physiology and disease. Nat Rev Cardiol. 2025;22(12):923–43. 40346347 10.1038/s41569-025-01162-x

[38] Bargieł W, Cierpiszewska K, Maruszczak K, Pakuła A, Szwankowska D, Wrzesińska A, et al. Recognized and potentially new biomarkers-their role in diagnosis and prognosis of cardiovascular disease. Medicina Kaunas. 2021. 10.3390/medicina57070701. 34356982 10.3390/medicina57070701 PMC8305174

[39] Gurung RL, Liu S, Liu JJ, M Y, Zheng H, Chan C, et al. Association of plasma angiogenin with risk of major cardiovascular events in type 2 diabetes. Cardiovasc Diabetol. 2024;23(1):70. 38360721 10.1186/s12933-024-02156-8 PMC10870605

[40] Ismail A, Hayek SS. Role of soluble urokinase-type plasminogen activator receptor in cardiovascular disease. Curr Cardiol Rep. 2023;25(12):1797–810. 37948017 10.1007/s11886-023-01991-7 PMC13501654

[41] LeBleu VS, Teng Y, O’Connell JT, Charytan D, Müller GA, Müller CA, et al. Identification of human epididymis protein-4 as a fibroblast-derived mediator of fibrosis. Nat Med. 2013;19(2):227–31. 23353556 10.1038/nm.2989 PMC4457508

[42] Khan H, Zamzam A, Shaikh F, Mamdani M, Saposnik G, Qadura M. HE4 as a prognostic biomarker of major adverse cardiovascular events in patients with abdominal aortic aneurysm: a Canadian prospective observational study. Biomedicines. 2025. 10.3390/biomedicines13071562. 40722638 10.3390/biomedicines13071562 PMC12292541

[43] Carey A, Parodi-Rullan R, Vazquez-Torres R, Canepa E, Fossati S. Homocysteine potentiates amyloid β -induced death receptor 4- and 5-mediated cerebral endothelial cell apoptosis, blood brain barrier dysfunction and angiogenic impairment. Aging Cell. 2024;23(5):e14106. 38358083 10.1111/acel.14106 PMC11113365

[44] Olszanecka A, Dragan A, Kawecka-Jaszcz K, Fedak D, Czarnecka D. Relationships of insulin-like growth factor-1, its binding proteins, and cardiometabolic risk in hypertensive perimenopausal women. Metabolism. 2017;69:96–106. 28285656 10.1016/j.metabol.2017.01.005

[45] Russo VC, Azar WJ, Yau SW, Sabin MA, Werther GA. IGFBP-2: the dark horse in metabolism and cancer. Cytokine Growth Factor Rev. 2015;26(3):329–46. 25544066 10.1016/j.cytogfr.2014.12.001

[46] POLR2F protein expression summary - The Human Protein Atlas. https://www.proteinatlas.org/ENSG00000100142-POLR2F

