# Supplementary material for: Chronic kidney disease onset, progression, and cardiovascular outcomes: proteomics informs biology and risk stratification
Source: Cardiovasc Diabetol. 2026 Jan 20;25:51. doi: 10.1186/s12933-025-03049-0 (PMC12903537; doi:10.1186/s12933-025-03049-0)
Supplement: Supplementary file 1 — Additional file 1 [file 12933_2025_3049_MOESM1_ESM.docx]

**Jijuan Zhang, et al, Chronic Kidney Disease Onset, Progression, and** **Cardiovascular Outcomes: Proteomics Informs Biology and Risk Stratification**

**Supplementary Methods.**

**Supplementary Figure S1.** Flow chart of the study.

**Supplementary Figure S2.** Shared and outcome-specific proteins across non-stroke outcomes.

**Supplementary Figure S3.** Top three enriched pathways of proteins associated with chronic kidney disease.

**Supplementary Figure S4.** Top three enriched pathways of proteins associated with end stage kidney disease.

**Supplementary Figure S5.** Top three enriched pathways of proteins associated with coronary heart disease.

**Supplementary Figure S6.** Top enriched pathways of proteins associated with stroke.

**Supplementary Figure S7.** Top three enriched pathways of proteins associated with heart failure.

**Supplementary Figure S8.** Calibration curves of predictive models for (A) chronic kidney disease and (B) end stage kidney disease.

**Supplementary Figure S9.** Calibration curves of predictive models for (A) coronary heart disease, (B) stroke, and (C) heart failure.

**Supplementary Figure S10.** Decision curves of predictive models for (A) chronic kidney disease and (B) end stage kidney disease.

**Supplementary Figure S11.** Decision curves of predictive models for (A) coronary heart disease, (B) stroke, and (C) heart failure.

**Supplementary Methods.**

**Study population**

In this study, for prospectively analyzing the associations between protein levels and the risk of chronic kidney disease (CKD), we initially included 53,017 participants with proteomic data, after excluding individuals with baseline CKD or those lacking data on estimated glomerular filtration rate or urinary albumin-to-creatinine ratio at baseline (n=8,238), we finally included 44,779 participants. For the prospective associations of plasma proteins with end stage kidney disease (ESKD), coronary heart disease (CHD), stroke, and heart failure (HF), we firstly included participants who had proteomic data and baseline CKD (n=4,455). After excluding those with baseline ESKD (n=287), CHD (n=706), stroke (n=241), and HF (n=183), respectively, we included 4,168, 3,749, 4,214, and 4,272 participants with prevalent CKD for the respective analyses of ESKD, CHD, stroke, and HF.

Baseline CKD was identified by an estimated glomerular filtration rate < 60 mL/min/1.73 m^2^, a urinary albumin-to-creatinine ratio ≥ 30 mg/g, self-reported history of CKD, or documentation of CKD in primary care or hospital admission records. Baseline ESKD was determined by an estimated glomerular filtration rate < 15 mL/min/1.73 m^2^, self-reported history of ESKD, or records of ESKD in primary care or hospital admission data. Baseline CHD, stroke, and HF were identified through self-reports or medical histories in primary care or hospital admission records.

**Covariates**

At baseline, comprehensive data were collected through questionnaires, physical assessments, and sample analyses. The Townsend deprivation index measures regional socioeconomic status, with higher values indicating greater deprivation. A healthy diet score, ranging from 0 to 10, was constructed based on intakes of ten food groups, with scores of 5 or higher indicating a healthy diet.^1^ Body mass index was calculated as weight (kg) divided by height squared (m^2^). Adequate physical activity was defined as at least 150 minutes of moderate-intensity exercise per week, 75 minutes of vigorous-intensity exercise per week, or a combination of both. The urinary albumin-to-creatinine ratio was calculated as the ratio of urinary microalbumin levels (mg/L) to urinary creatinine levels (g/L). The estimated glomerular filtration rate was calculated using the 2021 Chronic Kidney Disease Epidemiology Collaboration creatinine equation.^2^ A history of cardiovascular disease (including coronary heart disease, stroke, and heart failure) was identified through self-reported diagnoses or medical records. Hypertension history was assessed via self-report, use of antihypertensive medications, systolic/diastolic blood pressure ≥ 140/90 mmHg, or medical records. Diabetes history was identified through self-report, use of antidiabetic medications, fasting blood glucose ≥ 7 mmol/L, random blood glucose ≥ 11 mmol/L, glycated hemoglobin ≥ 48 mmol/mol, or medical records. Dyslipidemia history was determined based on self-report, use of lipid-lowering medications, triglyceride ≥ 1.7 mmol/L, high-density lipoprotein cholesterol < 1.03 mmol/L in men or < 1.29 mmol/L in women, or medical records.

**Reference:**

1. Said MA, Verweij N, van der Harst P. Associations of Combined Genetic and Lifestyle Risks With Incident Cardiovascular Disease and Diabetes in the UK Biobank Study. JAMA Cardiol. 2018;3:693-702.

2. Inker LA, Eneanya ND, Coresh J et al. New Creatinine- and Cystatin C-Based Equations to Estimate GFR without Race. N Engl J Med. 2021;385:1737-1749.


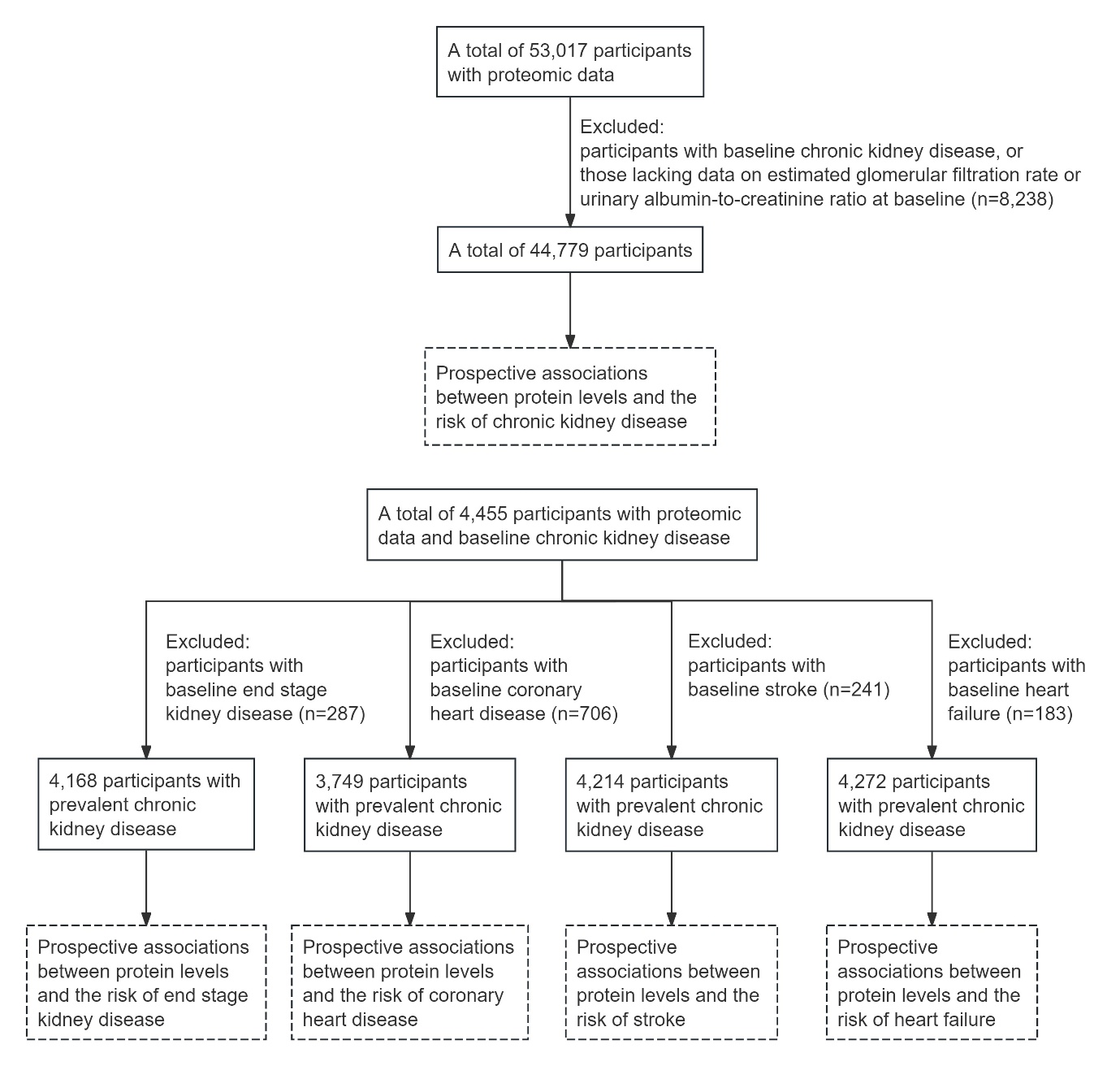


**Supplementary Figure S1.** Flow chart of the study.

**
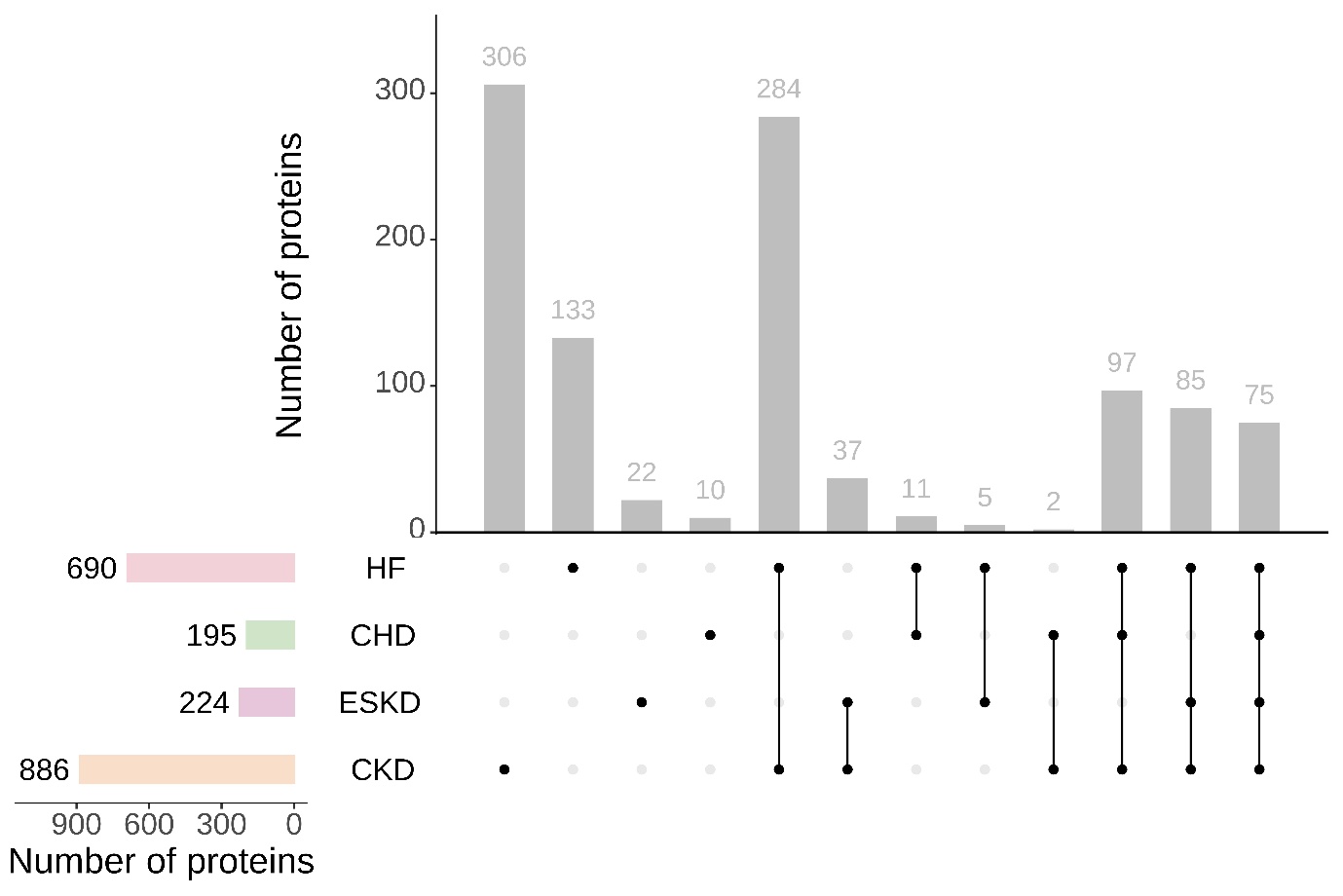
Supplementary Figure S2.** Shared and outcome-specific proteins across non-stroke outcomes. CHD, coronary heart disease. CKD, chronic kidney disease. ESKD, end stage kidney disease. HF, heart failure.

**
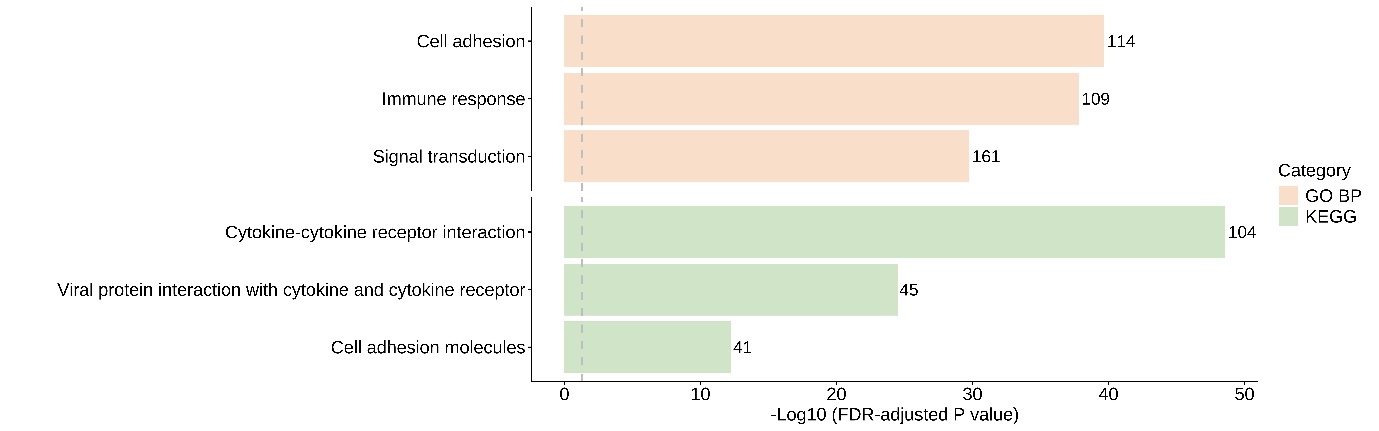
**

**Supplementary Figure S3.** Top three enriched pathways of proteins associated with chronic kidney disease. *P* values were calculated under two-sided tests and statistical significance was defined as a false discovery rate corrected *P* < 0.05 (dotted vertical line). The number near each bar is the number of observed proteins in each pathway. GO BP, Gene Ontology - biological process; KEGG, Kyoto Encyclopedia of Genes and Genomes; FDR, false discovery rate.


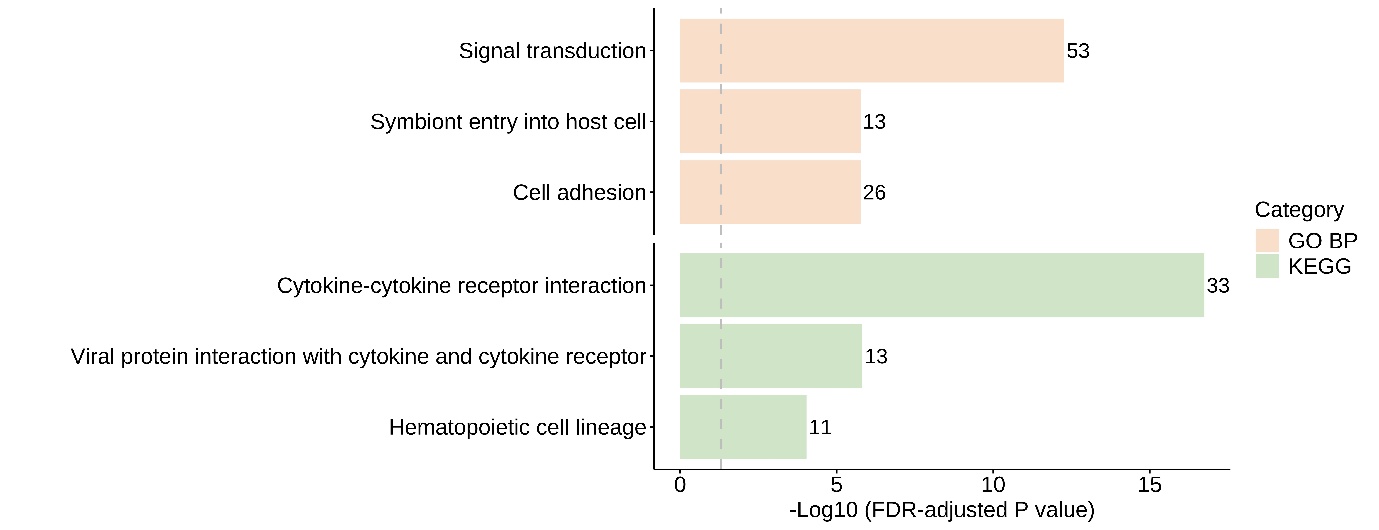


**Supplementary Figure S4.** Top three enriched pathways of proteins associated with end stage kidney disease. *P* values were calculated under two-sided tests and statistical significance was defined as a false discovery rate corrected *P* < 0.05 (dotted vertical line). The number near each bar is the number of observed proteins in each pathway. GO BP, Gene Ontology - biological process; KEGG, Kyoto Encyclopedia of Genes and Genomes; FDR, false discovery rate.


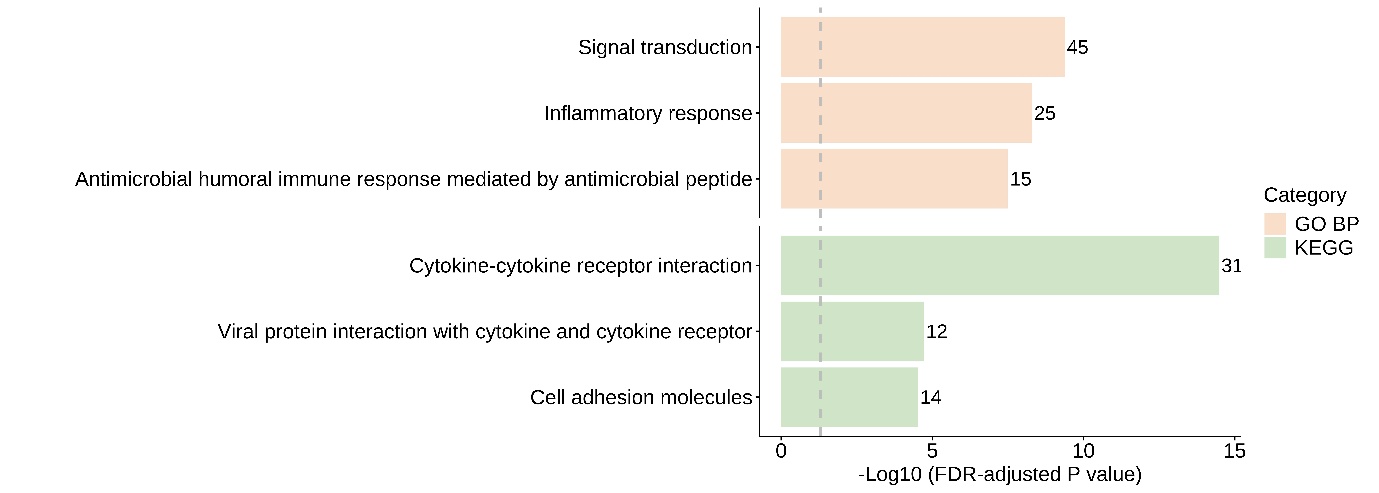


**Supplementary Figure S5.** Top three enriched pathways of proteins associated with coronary heart disease. *P* values were calculated under two-sided tests and statistical significance was defined as a false discovery rate corrected *P* < 0.05 (dotted vertical line). The number near each bar is the number of observed proteins in each pathway. GO BP, Gene Ontology - biological process; KEGG, Kyoto Encyclopedia of Genes and Genomes; FDR, false discovery rate.


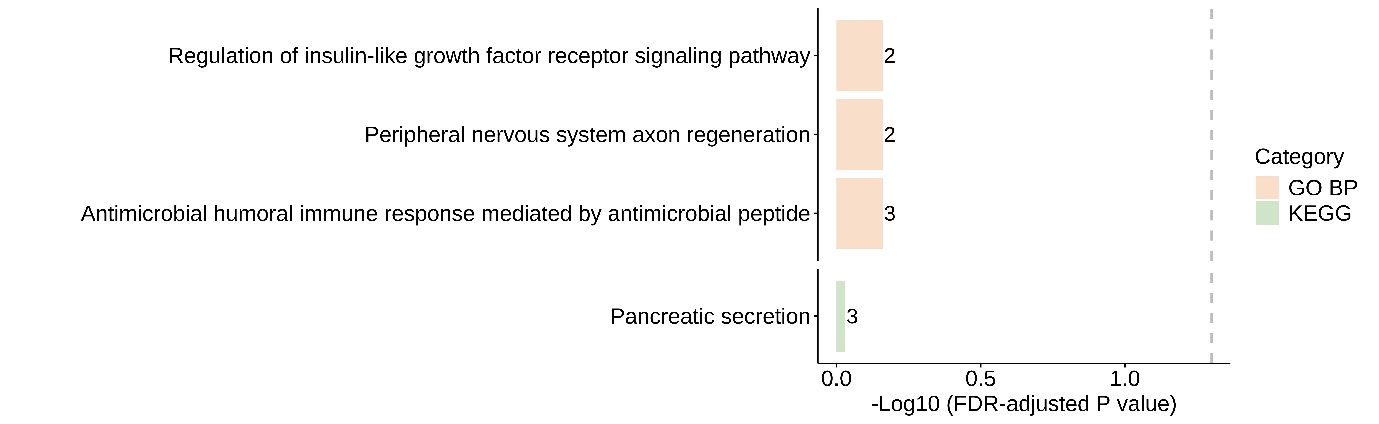


**Supplementary Figure S6.** Top enriched pathways of proteins associated with stroke. *P* values were calculated under two-sided tests and statistical significance was defined as a false discovery rate corrected *P* < 0.05 (dotted vertical line). The number near each bar is the number of observed proteins in each pathway. GO BP, Gene Ontology - biological process; KEGG, Kyoto Encyclopedia of Genes and Genomes; FDR, false discovery rate.


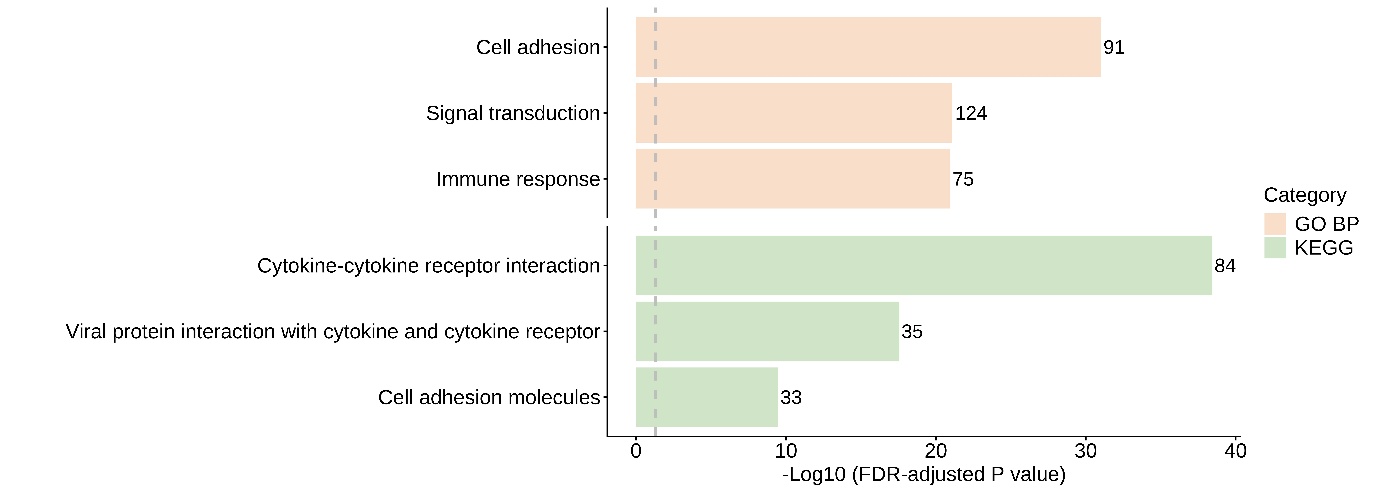


**Supplementary Figure S7.** Top three enriched pathways of proteins associated with heart failure. *P* values were calculated under two-sided tests and statistical significance was defined as a false discovery rate corrected *P* < 0.05 (dotted vertical line). The number near each bar is the number of observed proteins in each pathway. GO BP, Gene Ontology - biological process; KEGG, Kyoto Encyclopedia of Genes and Genomes; FDR, false discovery rate.

**
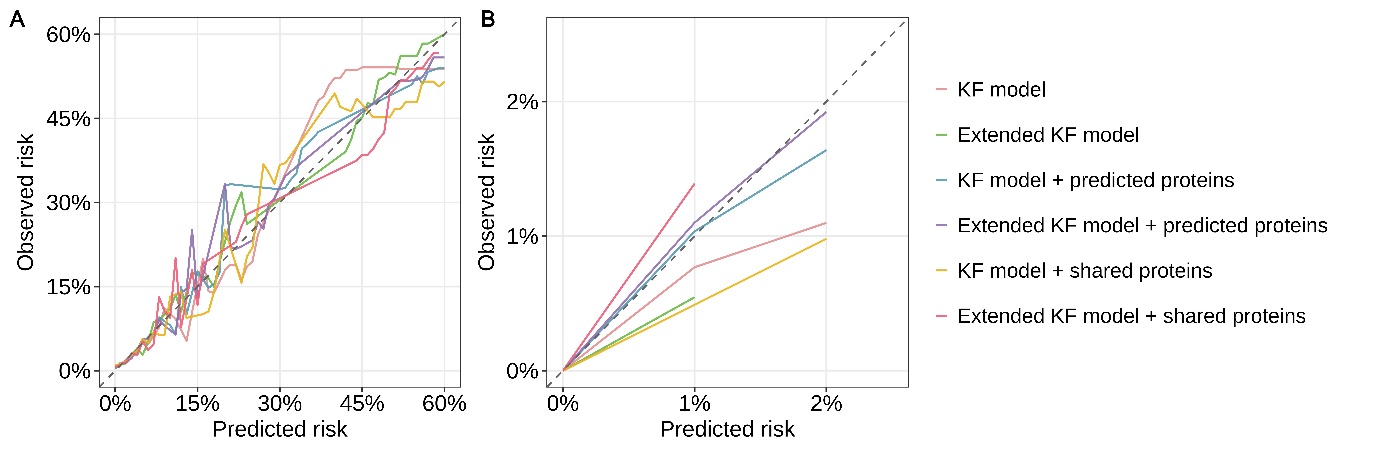
Supplementary Figure S8.** Calibration curves of predictive models for (A) chronic kidney disease and (B) end stage kidney disease. KF, kidney failure.

**
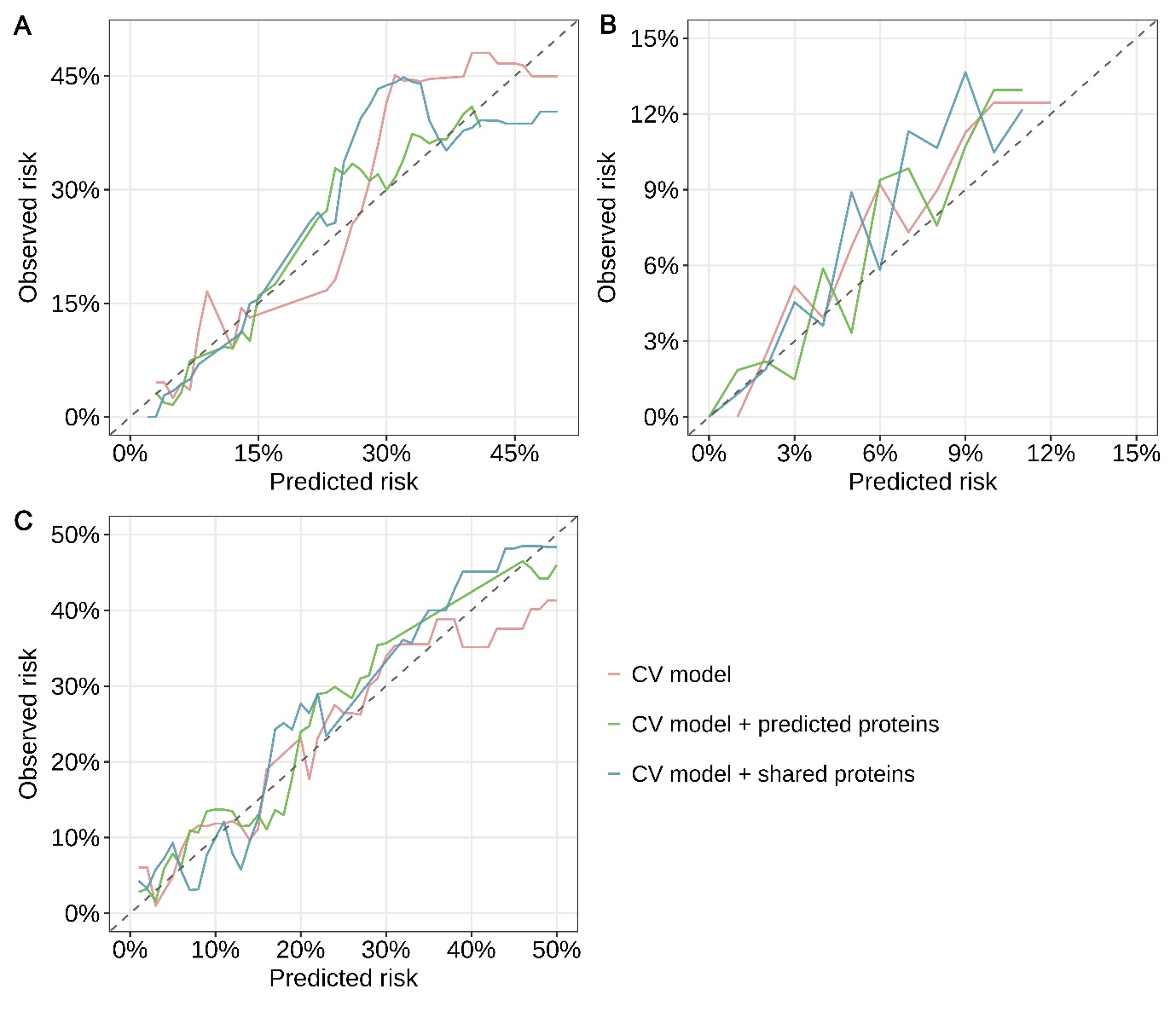
Supplementary Figure S9.** Calibration curves of predictive models for (A) coronary heart disease, (B) stroke, and (C) heart failure. CV, cardiovascular.

**
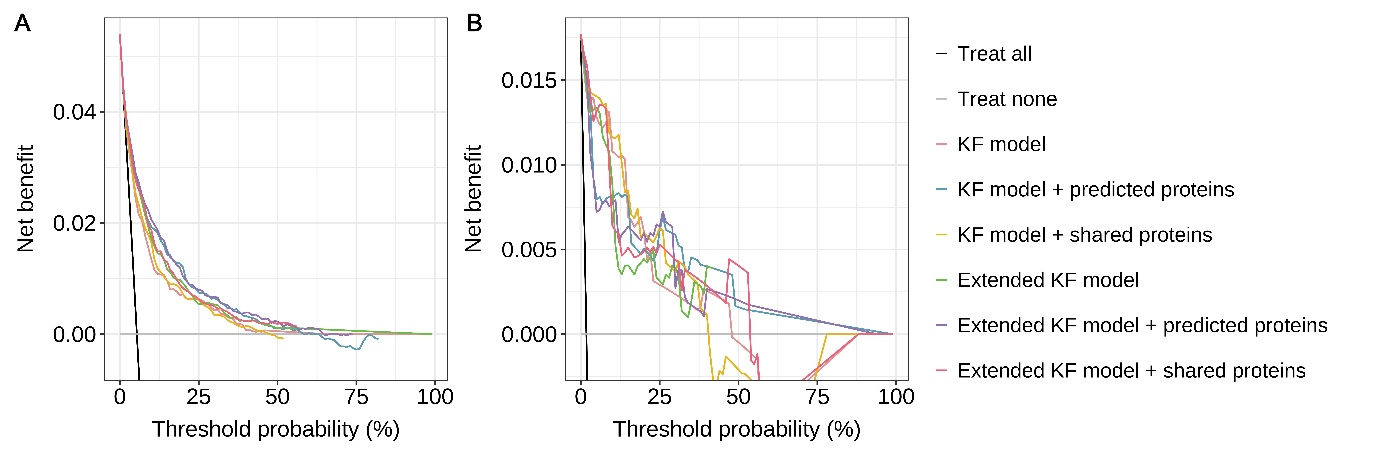
Supplementary Figure S10.** Decision curves of predictive models for (A) chronic kidney disease and (B) end stage kidney disease. KF, kidney failure.

**
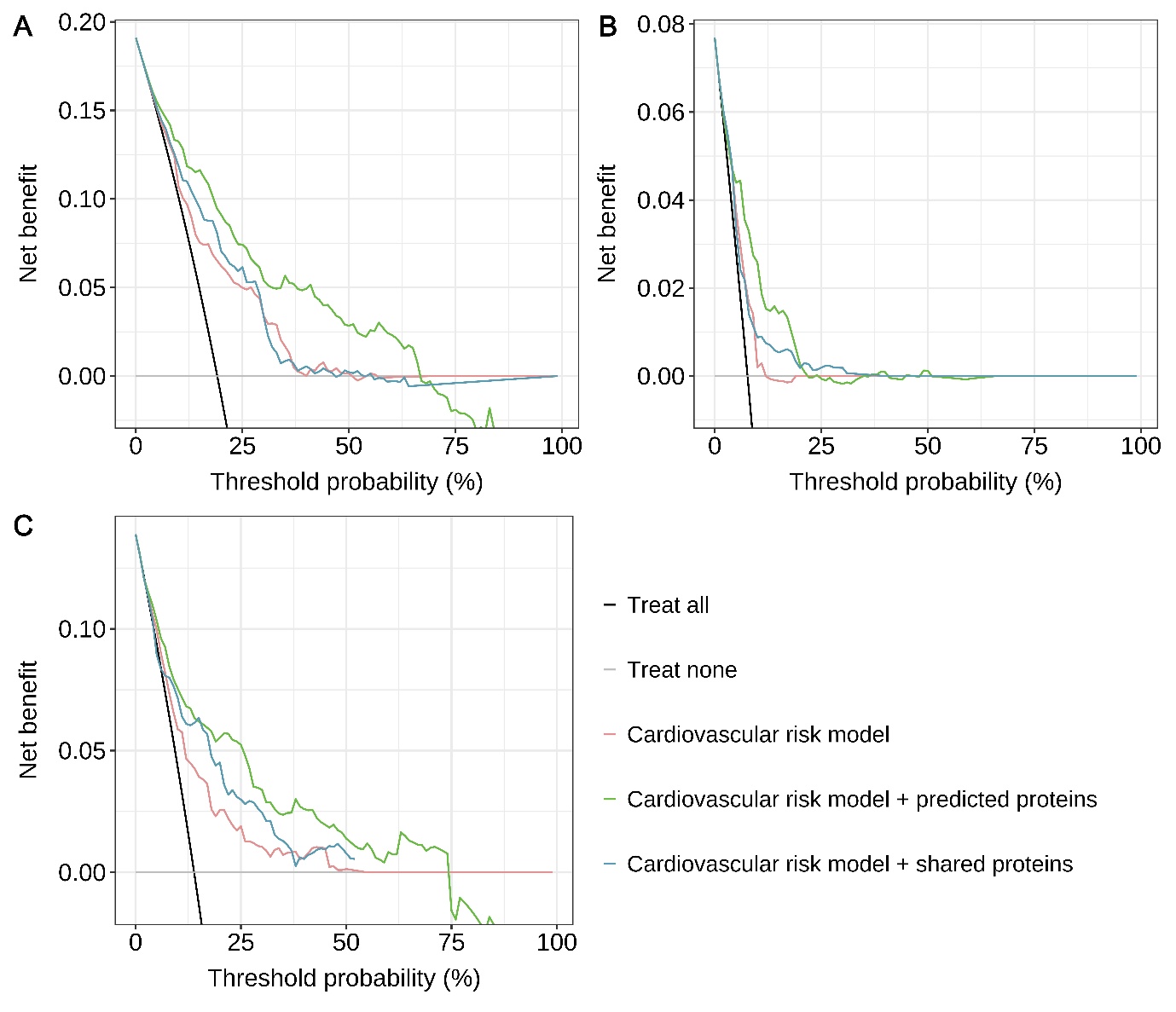
Supplementary Figure S11.** Decision curves of predictive models for (A) coronary heart disease, (B) stroke, and (C) heart failure.
